# Clinical diagnosis in paediatric patients at urban primary health care facilities in southern Malawi: a longitudinal observational study

**DOI:** 10.1186/s12913-021-06151-7

**Published:** 2021-02-15

**Authors:** Mtisunge Joshua Gondwe, Marc Y. R. Henrion, Thomasena O’Byrne, Clemens Masesa, Norman Lufesi, Queen Dube, Maureen D. Majamanda, Martha Makwero, David G. Lalloo, Nicola Desmond

**Affiliations:** 1 Behaviour and Health group, Malawi-Liverpool-Wellcome Trust Clinical Research Programme, Blantyre, Malawi; 2 Department of Clinical Sciences, Liverpool School of Tropical Medicine, Pembroke Place, Liverpool, L3 5QA UK; 3 Statistical Support Unit, Malawi-Liverpool-Wellcome Trust Clinical Research Programme, Blantyre, Malawi; 4 Trinity College, Dublin, Ireland; 5 Data Support Unit, Malawi-Liverpool-Wellcome Trust Clinical Research Programme, Blantyre, Malawi; 6 Department of Clinical services, Ministry of Health, Lilongwe, Malawi; 7 Department of paediatrics, Queen Elizabeth Central Hospital, Blantyre, Malawi; 8 Department of Medical and Surgical Nursing, University of Malawi, Kamuzu College of Nursing, Blantyre, Malawi; 9 Consortium for Advanced Research Training in Africa (CARTA), Nairobi, Kenya; 10 Department of Family Medicine, University of Malawi, College of Medicine, Blantyre, Malawi; 11 Department of International Public Health, Liverpool School of Tropical Medicine, Liverpool, UK

**Keywords:** ETAT, Triage, Primary health Centre, Paediatrics, Diagnosis, Stabilisation, Emergency, mHealth

## Abstract

**Background:**

Despite health centres being the first point of contact of care, there are challenges faced in providing care to patients at this level. In Malawi, service provision barriers reported at this level included long waiting times, high numbers of patients and erratic consultation systems which lead to mis-diagnosis and delayed referrals. Proper case management at this level of care is critical to prevent severe disease and deaths in children. We aimed to adopt Emergency, Triage, Assessment and Treatment algorithm (ETAT) to improve ability to identify severe illness in children at primary health centre (PHC) through comparison with secondary level diagnoses.

**Methods:**

We implemented ETAT mobile Health (mHealth) at eight urban PHCs in Blantyre, Malawi between April 2017 and September 2018. Health workers and support staff were trained in mHealth ETAT. Stabilisation rooms were established and equipped with emergency equipment. All PHCs used an electronic tracking system to triage and track sick children on referral to secondary care, facilitated by a unique barcode. Support staff at PHC triaged sick children using ETAT Emergency (E), Priority (P) and Queue (Q) symptoms and clinician gave clinical diagnosis. The secondary level diagnosis was considered as a gold standard. We used statistical computing software R (v3.5.1) and used exact 95% binomial confidence intervals when estimating diagnosis agreement proportions.

**Results:**

Eight-five percentage of all cases where assigned to E (9.0%) and P (75.5%) groups. Pneumonia was the most common PHC level diagnosis across all three triage groups (E, P, Q). The PHC level diagnosis of trauma was the most commonly confirmed diagnosis at secondary level facility (85.0%), while a PHC diagnosis of pneumonia was least likely to be confirmed at secondary level (39.6%). The secondary level diagnosis least likely to have been identified at PHC level was bronchiolitis 3 (5.2%). The majority of bronchiolitis cases (*n* = 50; (86.2%) were classified as pneumonia at the PHC level facility.

**Conclusions:**

Implementing a sustainable and consistent ETAT approach with stabilisation and treatment capacity at PHC level reinforce staff capacity to diagnose and has the potential to reduce other health system costs through fewer, timely and appropriate referrals.

## Background

Despite progress made in reducing childhood mortality, 1 in 13 children continued to die before their fifth birthday in Sub-Saharan Africa (SSA) in 2019 [1]. Though effective prevention and treatment interventions are available, complications during birth, pneumonia, diarrhoea, and malaria remain the biggest killers of under-five children in SSA [1]. In 2017, Malawi’s infant and under-five mortality rates were 42 and 63 deaths per 1000 live births respectively [2].

Primary health centres (PHC) are often the first point of contact within the health care system. Proper case management for patients is critical to prevent complications and death at this facility level. Some childhood deaths could be prevented if critically ill children were identified quickly at PHC, treated without delay and promptly referred to a secondary level hospital. There is evidence that quality primary health care reduces healthcare costs and increases efficacy by reducing hospital admissions [3].

Despite the fact that PHC are the first contact of care, there are frequent challenges in providing high quality care at this level [4–6]. Studies conducted in low and middle income countries (LMICs) have identified challenges experienced by staff working in government owned health centres. These include heavy patient workloads, inability to recognise severe illness, inadequate supervision, and limited clinical case management capacities [4, 5, 7–10]. In many low-income countries, less qualified personnel are often utilised to provide promotive, preventive and curative services across primary care settings [4, 11, 12].

Malawi’s health care system is organised at four levels, community, primary, secondary and tertiary [13]. The different levels are linked to each other through an established referral system. Health services are provided through the free public sector alongside private for profit and not for profit providers who charge a fee. The main provider for private not for profit services is faith-based hospitals, which provides approximately 29% of all health services in Malawi [13]. Faith-based hospitals are located in rural areas and have a Service Level Agreement (SLA) with government to provide maternal and child health services for free and later claim the cost from the public district hospital within their catchment area [13].

At community level, promotive and preventive health services are provided by health surveillance assistants (HSAs) through door-to-door visitations, village, outreach and mobile clinics. At primary level, health services are provided by health centres and community hospitals. Community hospitals are larger than health centres and offer both outpatient and inpatient care. Promotive, preventive, and curative health services are provided free of charge. Secondary level care provides both outpatient and inpatient services and consists of district and faith based hospitals receiving patients referred from both PHCs and community hospitals. Tertiary services in Malawi consist of four central hospitals which provide specialised care at regional level, receiving referrals from district hospitals within the region and where necessary wider field.

Queen Elizabeth Central Hospital (QECH), the largest referral hospital in Malawi, functions as both a secondary level facility for health centres in Blantyre district and as a tertiary hospital for the southern region of Malawi. Specialities offered at QECH include, medical, surgical, paediatric, neonatology, orthopaedic, oncology, ophthalmology, dermatology and Ear, Nose and Throat (ENT). Patients are referred from 29 public PHC as well as dispensaries managed by the Blantyre District Health Office. Additional services in the district are provided by private hospitals and clinics but the majority of the population of 1,251,484 [14] rely on the government and public services.

In Malawi, health centres provide mainly outpatient and maternity services. Health centres are meant to serve a population of 10,000. They are generally managed by medical assistants, clinical technicians and nurses with average training durations of 2–3 years [13]. Malawi suffers an acute shortage of qualified personnel with 1.49 health workers (clinical, nursing and allied staff) per 1000 population [5], far from the World Health Organisation (WHO) recommended ratio of 4.45 health workers per 1000 population [6]. As such many PHC staff in Malawi are trained to base their diagnosis and treatment on case management strategies. Some of these strategies used in Malawi by PHC health workers are Integrated Management of Childhood Illness (IMCI), Severe Acute Malnutrition (SAM) and Malaria guidelines. PHC health workers are trained on these guidelines and largely base their clinical decisions on them.

Over half of Blantyre district population is under 15 years old [15] and a high proportion of presentations at primary level are for paediatric services. Burdens at individual clinics are high with a health facility per population ratio for the city exceeding the recommended urban planning standard of 10,000 persons per facility [6, 15]. In addition, health centres in the district are under-staffed and lack adequate resources, leading to many paediatric deaths occurring within the first 24 h of admission [16–18]. At PHCs, standard practice has been for adults and children to queue together and to be seen on a ‘first come, first served basis’. Triage at PHCs was rare before implementation of ETAT and severe illness in children was often missed, resulting in mortality, disability and complications [10], often increasing burdens on primary facilities.

**Fig. 1:**
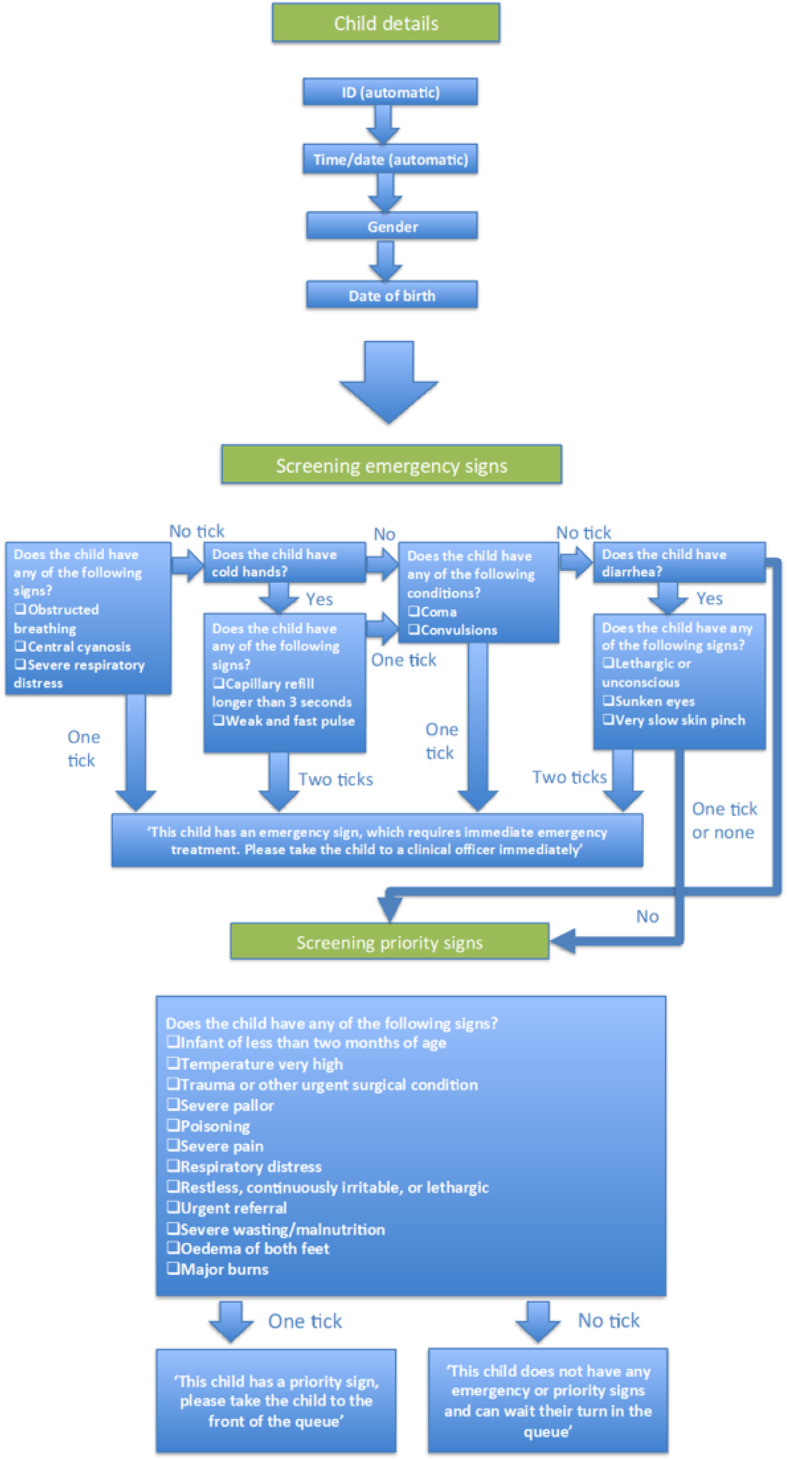
Emergency Triage Assessment and Treatment (ETAT) mHealth Algorithm

Similar problems with diagnosis have been recognised at tertiary level, driving the QECH paediatric department team to implement the triage concept to improve emergency care in 2001 [19]. Later then, the team lead by Professor Elizabeth Molyneux developed the Emergency Triage Assessment and Treatment (ETAT) guidelines in collaboration with the WHO in 2005 [20, 21]. These guidelines have since been adopted across other LMIC settings. The ETAT guidelines were adapted from the Advanced Paediatric Life Support guidelines used in high income countries. They identify children with immediately life-threatening conditions most frequently seen in LMIC, such as obstruction of the airway and other breathing problems caused by infections, shock, severely altered central nervous system function (coma or convulsions), and severe dehydration [22]. The ETAT guidelines are designed to identify sick children soon after their arrival in the facility in order to start emergency treatment immediately to reduce mortality [22]. The guidelines follow the ABCD concept (Airway-Breathing-Circulation-Coma-Convulsion-Dehydration) and priority signs as shown in Fig. 1. These are an integral part of the IMCI system to reduce childhood illness [23]. Following the success of integrating ETAT into health programmes to improve paediatric triage training in 8 tertiary and secondary level hospitals [22], ETAT has since been introduced to more than 54 hospitals in Malawi, including QECH, resulting in a process of rapid triage for all children to determine whether any emergency or priority signs are present, followed by prompt emergency treatment [24, 25].

ETAT has been delivered at primary level in some rural districts of Malawi, but it is evident that it is urgently required in urban health care settings, specifically Blantyre, which has some of the busiest health centres in Malawi, and no district hospital to share the burden. We introduced an electronic algorithm for consistent implementation of ETAT in urban health centres in 2013, where ETAT had not yet been introduced. Since then, the triage component of ETAT was implemented until 2017, when we added the full ETAT package including emergency management.

Following implementation of the full ETAT protocol at PHC level in Blantyre district using the mHealth algorithm to improve consistency in diagnosis this study was designed to expand on the primary level intervention to document the capacity of PHC level staff to diagnose severity of illness, stabilise patients and make appropriate and timely referral decisions. As such this paper provides a useful contribution to understanding accuracy of severe illness identification and ways of strengthening clinical diagnosis at PHC level facility.

## Methods

### Study area and study centres

The analysis in this paper focuses on eight urban PHCs in Blantyre district where a full tracking system was operated. The catchment population of the PHCs was 795,384 of which 5, 17 and 48% were under one, five and 15 years old respectively in 2017 [15]. All eight PHCs were primarily staffed by clinical technicians or medical assistants and nurses with an average training duration of 2–3 years. Nurses dealt largely with primary maternal and child health services while clinical technicians or medical assistant’s dealt with outpatient services. HSA’s roles generally link PHCs with the community through the provision of preventive and promotive services, but they are increasingly taking on basic clinical roles such as Human Immunodeficiency Virus (HIV) and Tuberculosis (TB) care in PHCs due to staff shortages. Hospital attendants are non-technical staff, who assist with cleaning. Clinical technicians or Medical assistants at PHC treat uncomplicated common childhood illness that does not need inpatient care. For childhood, the diagnosis and treatment plan are mainly based on the Integrated Management of Childhood Illness (IMCI) and Malaria guidelines [13]. Conditions treated at this level include, uncomplicated malaria, pneumonia, Acute Respiratory Infections (ARIs), diarrhoea disease and other mild illnesses [13]. The capacity to perform laboratory investigations is limited, supporting only basic investigations like malaria diagnostic tests (rapid and microscopy tests), haemoglobin level, syphilis testing, TB screening and HIV tests [13].

### The intervention context

#### The mHealth triage algorithm

An mHealth phone-based triage algorithm was developed in collaboration with D-Tree International, the Ministry of Health and the Data Department from Malawi Liverpool Wellcome Trust Clinical Research Programme (MLW). This algorithm is based on the ETAT triage component for tertiary level developed in collaboration with the WHO. This follows the ABCD concept and priority signs as shown in algorithm in Fig.1. Available on a mobile phone device in order to promote mobility of staff within busy clinic waiting rooms, staff responsible for triage worked through a series of simple questions to assess the level of priority for each child attending the clinic. Priority status is established within 20–30 s using the algorithm. Basic socio-demographic data including assignment of a Personal Identification Number (PID), age and sex were assigned for each patient along with automatically assigned data including PHC, time and date. Individual Health Care Workers (HCW) input their ID on first using the phone each day.

#### Implementation of the intervention

We implemented ETAT-based triage with mHealth technology to prompt healthcare workers (nurses, clinicians and some allied staff) and support staff (drivers, clerks, HSAs, patient attendants, hospital attendants and hospital guards) to recognise and prioritise children with serious illness. Through this project the national ETAT training manual was revised to better align with the needs of PHC level staff. Clinicians and nurses were trained in mHealth triage and emergency management for 2.5 days while support staff were trained in mHealth triage for 1.5 days. In addition, rooms were established and equipped with emergency drugs and supplies to ensure that patients with emergency signs could be stabilised before referral. All PHCs were supplied with mobile phones. No personal identifiers of patients were collected on the mHealth phones. A unique barcode was stamped into each patient’s health passport to trace individual experiences through the system and to facilitate linkage between different sites within the study. Each barcode was linked to date of birth, age, sex, the PHC level facility and outcome for data analysis. The health passport is a government booklet used by HCW to record illness events each time a child or patient visits any health facility.

### Data and study population

**Fig. 2:**
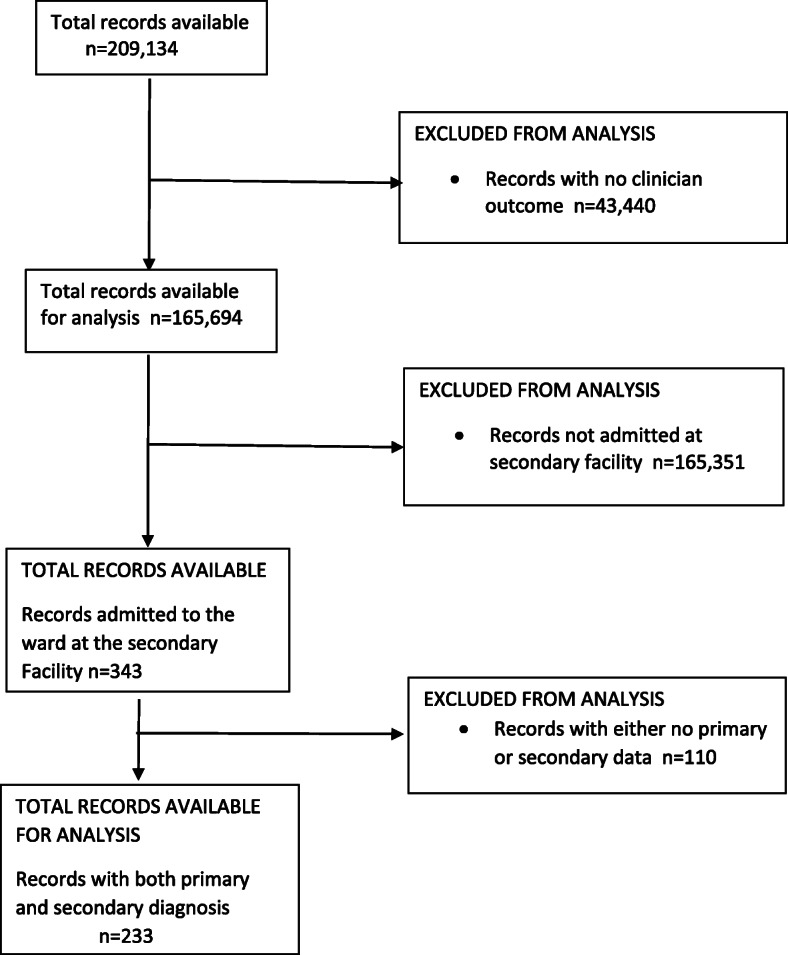
Study Population

The study population comprised all paediatric patients (*n* = 209, 134) aged 0–14 years seeking care in any of the eight PHC outpatient departments (OPD) between April 2017 and September 2018 (Fig. 2). Paediatric definitions of 0 to 14 were based on those defined at central hospitals. Records without clinician outcome data from the PHCs (*n* = 43,440) and those not admitted at secondary facility *n* = 165,351 were removed from the analysis. A further 110 records were excluded as they had either no PHC or secondary level data, leaving 233 records for the analysis (Fig. 2).

### Data collection, diagnosis and stabilisation

Data were collected using mHealth phones at four stages. The first stage was upon arrival at the waiting area in a paediatric outpatient department in PHCs where patients were triaged by support staff who placed a barcode in the health passport and assigned a prioritisation group (Emergency (E), Priority (P) and Queue (Q)). Children assigned to the Emergency group were taken for immediate consultation or to the resuscitation room for emergency treatment, those assigned to the Priority group were moved to the front of the queue and those with a Queue assessment awaited their turn to be seen by the clinician. At the second stage, clinicians verified the triage category. A fieldworker was posted at each PHC to capture clinician triage and consultation outcome and referral information through a second mobile phone. At the third stage, a mobile phone was again used at the outpatient department of the secondary level facility (QECH) if arrival was between 7:30 am and 10 pm. All patients (0–14) arriving at QECH had their health passports and referral stamp checked by a fieldworker, who scanned the barcode sticker using the phone to retrieve the patient’s details and verify that the patient had arrived at QECH. Finally, at the fourth stage, a research nurse scanned the barcode using a tablet to link the triage category and PHC data to QECH admissions data and to retrospectively collect information on patient diagnosis. The secondary level diagnosis from QECH is considered to be the gold standard diagnosis in this paper, given that QECH has full diagnostic capacity through functional laboratories and is staffed with clinical consultants and registrars.

### Statistical analysis

The mHealth data were stored on a central, access-controlled, encrypted database. Statistical analyses were performed using the statistical computing software R (v3.5.1) [26]. We computed descriptive statistics (i.e. frequencies, percentages) and used exact 95% binomial confidence intervals when estimating diagnosis agreement proportions.

## Results

### Participant characteristics

Table 1 lists the sex, age and triage distribution of patients captured by the 233 analysed records. There are more male (45.1%) children than female (31.8%) and the majority were under-five (57.9%).

**Table 1:** Personal characteristics of successful referrals who had both PHC and secondary diagnosis captured (*n* = 233)

Patient characteristics	n	(%)
**Sex**		
Female	74	(31.8%)
Male	105	(45.1%)
Not recorded	54	(23.2%)
**Age**		
Neonates and infants (< 12 months)	76	(32.6%)
12–59 months	59	(25.3%)
5 years and above	44	(18.9%)
Not recorded	54	(23.2%
**PHC level triage**		
Emergency (E)	21	(9.0%)
Priority (P)	176	(75.5%)
Queue (Q)	36	(15.5%)

### Triage category at PHC

Out of 233 records the most frequent ETAT triage categorisation was assigned to priority (P) cases, followed by non-urgent cases (Queue) then emergency cases (E) across all 8 facilities (Table 1).

### Agreement between PHC and secondary diagnoses

Agreement between PHC and secondary diagnosis needs to be looked at from two directions: i) the proportion of PHC diagnoses that are confirmed at secondary (this speaks to how likely a given PHC diagnosis is to be correct and can help to identify over diagnosed conditions) and ii) the proportion of secondary level diagnoses that were already identified at PHC (this speaks to which conditions are likely to be missed at PHC).

Generally, common childhood illnesses were over diagnosed at PHC level facilities (Table 2). Trauma and malnutrition were the PHC diagnoses with highest confirmation rates (over 80%) at secondary facility (Table 2). Pneumonia cases were commonly over-diagnosed at PHC facilities with only 39.6% of the diagnoses confirmed at QECH.

**Table 2:** ^a,b^ Agreement between PHC and secondary level facility on common childhood illness diagnosis

	PRIMARY HEALTH CENTRE				SECONDARY LEVEL FACILITY			
Diagnosis	Total	Confirmed at secondary n (%) [95% CI]			Total	Identified at primary n (%) [95% CI]		
**Anaemia**	24	19	(79.2%)	[57.9,92.9%]	25	19	(76.0%)	[54.9,90.6%]
**Bronchiolitis**	4	3	(75.0%)	[19.4,99.4%]	58	3	(5.2%)	[1.1,14.4%]
**GE**	12	9	(75.0%)	[42.8,94.5%]	23	9	(39.1%)	[19.7,61.5%]
**Malaria**	44	29	(65.9%)	[50.1,79.5%]	39	29	(74.4%)	[57.9,87.0%]
**Malnutrition**	6	5	(83.3%)	[35.9,99.6%]	18	5	(27.8%)	[9.7,53.5%]
**Meningitis**	9	4	(44.4%)	[13.7,78.8%]	7	4	(57.1%)	[18.4,90.1%]
**Pneumonia**	91	36	(39.6%)	[29.5,50.4%]	44	36	(81.8%)	[67.3,91.8%]
**Sepsis**	41	24	(58.5%)	[42.1,73.7%]	54	24	(44.4%)	[30.9,58.6%]
**Trauma**	20	17	(85.0%)	[62.1,96.8%]	18	17	(94.4%)	[72.7,99.9%]

^a^The total number of case records exceed 233 in this table as patients can be diagnosed with more than one condition (co-morbidity) at both PHC and secondary level facility

^b^In the Primary Health Centre columns, the denominator is the total number of diagnoses made at PHC for each condition, whereas in the Secondary Level Facility columns, the denominator is the total number of diagnoses made at the secondary level facility for each condition. The numerators for each set of columns are the same – the number of times a condition was diagnoses both at PHC and at the secondary level

### Identification of secondary diagnosis at PHC level facility

The likelihood of the secondary level facility diagnosis being correctly identified at PHC level clinics was high for trauma, pneumonia, anaemia and malaria cases with 94.4, 81.8, 76.0 and 74.4% respectively (Table 2). The secondary level diagnosis least likely to be identified was bronchiolitis with only 3 (5.2%) correctly identified at PHC.

### Co-occurrence of diagnosis at PHC and secondary level facilities

Table 3 lists how many times two given conditions were co-diagnosed at PHC and secondary level facility. To calculate percentages, we used the number of unique diagnoses at the secondary level. The majority of bronchiolitis cases at secondary level, 50 of 58 (86.2%), were diagnosed as pneumonia at PHC level while 16 of 25 (64.0%) of anaemia cases at secondary level were classified as malaria at PHC level. Almost all trauma cases 17 of 18 (94.44%) at secondary level were also classified as trauma at PHC level facility.

**Table 3:** ^a^ Proportions (number) distribution of secondary level diagnosis against PHC level diagnosis

Secondary level facility																		
	Anaemia		Bronchiolitis		GE		Malaria		Malnutrition		Meningitis		Pneumonia		Sepsis		Trauma	
**Anaemia**	76.00%	19	0.00%	0	4.35%	1	35.90%	14	11.11%	2	0.00%	0	2.27%	1	7.41%	4	0.00%	0
**Bronchiolitis**	0.00%	0	5.17%	3	4.35%	1	0.00%	0	0.00%	0	0.00%	0	0.00%	0	0.00%	0	0.00%	0
**GE**	0.00%	0	0.00%	0	39.13%	9	2.56%	1	5.56%	1	0.00%	0	0.00%	0	12.96%	7	0.00%	0
**Malaria**	64.00%	16	6.90%	4	30.43%	7	74.36%	29	22.22%	4	14.29%	1	6.82%	3	14.81%	8	0.00%	0
**Malnutrition**	0.00%	0	0.00%	0	0.00%	0	0.00%	0	27.78%	5	14.29%	1	4.55%	2	5.56%	3	0.00%	0
**Meningitis**	4.00%	1	0.00%	0	8.70%	2	2.56%	1	0.00%	0	57.14%	4	4.55%	2	11.11%	6	0.00%	0
**Pneumonia**	4.00%	1	86.21%	50	21.74%	5	7.69%	3	27.78%	5	14.29%	1	81.82%	36	18.52%	10	0.00%	0
**Sepsis**	12.00%	3	8.62%	5	17.39%	4	15.38%	6	22.22%	4	57.14%	4	11.36%	5	44.44%	24	5.56%	1
**Trauma**	0.00%	0	0.00%	0	0.00%	0	0.00%	0	0.00%	0	0.00%	0	0.00%	0	1.85%	1	94.44%	17
**Total unique diagnoses at Secondary level**		25		58		23		39		18		7		44		54		18

^a^The total number of case records exceed 233 in this analysis as patients can be diagnosed with more than one condition at both PHC and secondary level facility

## Discussion

In the implementation of mhealth ETAT at eight urban primary health centres in Malawi to improve consistency in diagnosis, a low proportion of emergency (E) cases were referred to the secondary level compared to priority (P) cases. Diagnosis of trauma, malnutrition and anaemia had high likelihood of being confirmed at secondary level facility. Pneumonia was over-diagnosed at PHC while bronchiolitis was underdiagnosed at PHC.

Campbell and colleagues [27] have argued that 10–20% of sick children presenting at PHC will need to be referred to the next level facility. The relatively low proportion of emergency cases referred to a secondary level facility in this study may be due to functional ETAT systems at PHCs, which improved stabilisation of children with serious illness that subsequently improved and reversed the need for referral. Furthermore, other emergency (E) cases may have bypassed the triage system and therefore were not included in the analysis as they would have been identified immediately pre-triage and are taken straight to the stabilisation room at the PHC so they would not necessarily be captured within our data monitoring system.

Children in Malawi continue to die from preventable causes such as sepsis, lower respiratory tract infection, gastroenteritis (GE), meningitis and malaria [28]. A high proportion of PHC diagnosis of trauma, malnutrition and anaemia was confirmed at secondary level which may be attributable to the unique clinical presentations of these conditions in comparison to more complex symptom presentation, facilitating early differentiation of cause amongst clinicians at PHC level. In contrast, clinicians at PHC often face challenges to correctly diagnose malaria, sepsis, meningitis and pneumonia commonly leading to under or misdiagnosis due to similar disease presentations. These often challenge clinicians to correctly diagnose and treat especially if diagnostic resources are stretched as is often the case at PHC level in LMICs [29]. Furthermore, due to limited resources, clinical technicians and medical assistants are often trained to manage symptoms. Despite case management guidelines and algorithms advocated to improve management, accurate diagnosis of these conditions has proven to be difficult due to their similar presentations [30]. A study in Malawi reports that 95% of children with a clinical case definition for pneumonia also meet the malaria case definition [31]. Furthermore the general clinicians at some PHC facilities are lowly trained compared to the tertiary hospital. Future analysis should compare urban versus rural facilities as that would also show the disparities in training levels.

Pneumonia and bronchiolitis are conditions with similar clinical presentation and differentiation has previously been reported as a challenge for clinicians in the absence of diagnostic tests [31, 32]. This may be particularly challenging for clinicians working in PHC as they use case management strategies such as the IMCI guidelines. A case management approach assumes that the presentation of fever and cough with fast breathing and/or chest indrawing is most likely due to pneumonia and is to be treated with a course of antibiotics [33]. Due to high numbers of patients and frequent unavailability of stethoscopes, clinicians at PHC are unlikely to undertake comprehensive chest assessments to accurately differentiate bronchiolitis from pneumonia. A study conducted in four hospitals in India found that many children who fulfil WHO’s traditional criteria for pneumonia (cough and difficulty breathing with or without chest indrawing) have wheezy viral infections [33]. WHO has recently updated its guidelines for the management of acute respiratory infections (ARIs) in children to include the differential diagnosis of cough and difficulty breathing, and separate guidelines for the management of pneumonia, bronchiolitis and asthma [34]. Failure to follow these guidelines, they warn, will lead to the overuse of antibiotics and under treatment of asthma both of which have significant public health implications in low resource settings such as Malawi [35].

Recognising meningitis at PHC is a recognised challenge [10, 36, 37] since it has overlapping clinical presentations with other febrile illnesses. The PHC staff in Malawi are trained to perform basic laboratory investigations such as rapid diagnostic tests for malaria and haemoglobin but are unable to perform lumbar puncture or collect cerebrospinal fluid (CSF) and full blood count analysis. However, reagents are often out of stock for even these basic investigations [38, 39].

This study has some limitations. The mHealth tracking system operated during weekdays from 7:30 am to 4:30 pm at PHCs and 7:30 am to 10 pm at secondary outpatient departments. This may mean some patients reporting after these hours were missed. The system did not capture patient visits outside these hours. Secondly the analysis did not include all patients who were referred and received a PHC diagnosis, as some did not arrive at the secondary facility while others arrived but were not admitted to the ward so the system did not capture their data. Thirdly the analysis only included 8 urban PHCs from a pool of district hospitals and both urban and rural primary clinics that refer directly to QECH within the Southern region of Malawi. As such, the numbers included are unlikely to be a true reflection of total patients admitted to QECH. Fourthly we have taken the QECH diagnosis as the gold standard for this comparative study but we acknowledge that this may not always be the case. However, given that QECH has full diagnostic capacity and highly trained clinicians it is likely that diagnosis is as optimal as feasible in this LMIC setting. In addition, data quality has been impacted the mhealth system as it did not capture clinician outcome data in 20% (*n* = 43,400) of the study population (*n* = 209,134). Furthermore, data on age and sex were not captured by the system in 23.2% (*n* = 54) of all records (*n* = 233) included in the final analysis. Though some of the reasons for the missing diagnoses were the study design given the working hours rather than an issue with data entry. The study, however, provides a useful contribution to understanding the accuracy of severe illness identification at primary level.

## Conclusions

This study provides a useful overview of the disparity in diagnoses between primary and secondary/tertiary level facilities, highlighting the knock-on effect of resource constraints at primary level facilities in LMIC. Implementing mhealth ETAT at urban primary health centres is possible, though the system had some issues with data capture that should be addressed in future implementation. The results revealed that pneumonia is over-diagnosed at PHC level, largely due to misdiagnosis of bronchiolitis or other Lower Respiratory Tract Infection (LRTI) with similar symptoms. While a tendency to over rather than under-diagnosis is preferable since this ensures patients with Upper Respiratory Tract Infection (URTI) are less likely to be missed, it places additional strains on both human and material resources within the health system, especially at secondary/tertiary level.

Having a functional formal system for consistent triage of sick children at PHC using established ETAT guidelines is essential to isolate very sick children presenting with emergency signs in order to prompt appropriate referral and treatment at secondary level.

However, to optimise benefits from the introduction of ETAT at PHC level it is important that it is well integrated with the Integrated Management of Childhood Illness (IMCI) initiative so as to improve overall patient management and outcomes. For health care workers at PHC to provide optimal care ETAT implementation should be integrated with regular training, case management guidelines, emergency supplies, equipment, and drugs.

## Data Availability

The datasets used and analysed during the current study are available from the corresponding author on reasonable request.

## Abbreviations

ETAT: Emergency Triage Assessment and Treatment
PHC: Primary Health Centre
mHealth: Mobile Health
E: Emergency
P: Priority
Q: Queue
SSA: Sub-Saharan Africa
LMIC: Lower-Middle-Income Country
SLA: Service Level Agreement
HSA: Health Surveillance Assistant
QECH: Queen Elizabeth Central Hospital
ENT: Ear, Nose and Throat
WHO: World Health Organization
IMCI: Integrated Management of Childhood Illness
SAM: Severe Acute Malnutrition
ABCD: Airway, Breathing, Circulation, Coma, Convulsion and Dehydration
HIV: Human Immunodeficiency Syndrome
TB: Tuberculosis
ARI: Acute Respiratory Infection
MLW: Malawi -Liverpool -Wellcome Trust, Clinical Research Programme
PID: Patient Identification Number
HCW: Health Care Worker
OPD: Out-Patient Department
GE: Gastroenteritis
CSF: Cerebrospinal Fluid
LRTI: Lower Respiratory Tract Infection
URTI: Upper Respiratory Tract Infection
ASPIRE: Achieving Sustainable Primary Improvement and Engagement in Health
PEAG: Primary ETAT Advisory Group
MRF: Meningitis Research Foundation

## References

[1] UNICEF, UN IGME (2020). Levels & Trends in Child mortality Report 2020.

[2] National Statistical Office (NSO) [Malawi] (2017). ICF Macro: Malawi Demographic and Health Survey 2015–16.

[3] World Health Organization (2018). Building the economic case for primary health care: a scoping review.

[4] Jaeger FN, Bechir M, Harouna M, Moto DD, Utzinger J (2018). Challenges and opportunities for healthcare workers in a rural district of Chad. BMC Health Serv Res.

[5] Resource H, Government of Rebublic of Malawi (2018). Human resources for Health strategic plan 2018–22.

[6] World Health O (2016). Health workforce requirements for universal health coverage and the sustainable Development goals. (human resources for Health observer, 17).

[7] Alhassan RK, Spieker N, van Ostenberg P, Ogink A, Nketiah-Amponsah E, de Wit TFR (2013). Association between health worker motivation and healthcare quality efforts in Ghana. Hum Resour Health.

[8] Topp SM, Chipukuma JM, Hanefeld J (2015). Understanding the dynamic interactions driving Zambian health Centre performance: a case-based health systems analysis. Health Policy Plan.

[9] Goetz K, Marx M, Marx I, Brodowski M, Nafula M, Prytherch H, Omogi Awour IKE, Szecsenyi J (2015). Working atmosphere and job satisfaction of Health care staff in Kenya: an exploratory study. Biomed Res Int.

[10] Desmond NA, Nyirenda D, Dube Q, Mallewa M, Molyneux E, Lalloo DG, Heyderman RS (2013). Recognising and treatment seeking for acute bacterial meningitis in adults and children in resource-poor settings: a qualitative study. PLoS One.

[11] Motherhood FS, Newborn Health C (2009). Human resources for health in the low-resource world: collaborative practice and task shifting in maternal and neonatal care. Int J Gynecol Obstet.

[12] World Health Organization (2007). Task shifting to tackle health worker shortages.

[13] Government of Republic of Malawi (2017). Health Sector Strategic Plan II (2017–2022). Ministry of Health.

[14] Office NS, National Statistical Office (2018). Malawi population and housing census: Main report.

[15] Blantyre District Health Office (2017). Health management information system 2017/2018. Blantyre.

[16] Hategeka C, Nshuti S, Mucumbitsi J, Muvunyi C, Mutesa L, Rusingiza E (2012). Management challenges of pediatric infective endocarditis at tertiary level in Rwanda. Rwanda Med J.

[17] Ralston ME, Day LT, Slusher TM, Musa NL, Doss HS (2013). Global paediatric advanced life support: improving child survival in limited-resource settings. Lancet.

[18] Farham B, Mabey D, Gill G, Parry E, Weber MW, Whitty CJM (2013). Principles of Medicine in Africa.

[19] Molyneux E, Ahmad S, Robertson A (2006). Improved triage and emergency care for children reduces inpatient mortality in a resource-constrained setting. Bull World Health Organ.

[20] Gray A, Maclennan C. What are the pre-requisites/pre-conditions for emergency triage and treatment (ETAT) to be beneficial? Int Child Health Rev Collaboration. 2008.

[21] College of Medicine UoM (2008). ETAT Malawi Project: Report and Evaluation.

[22] World Health Organization (2005). Emergency Triage Assessment and Treatment (ETAT)- Manual for Participants. Development DoCaAHa.

[23] Pollock L (2009). ETAT Malawi project: report and evaluation.

[24] Robison J, Ahmed Z, Durand C, Nosek C, Namathanga A, Milazi R, Thomas A, Mwansambo C, Kazembe PN, Torrey S (2011). Implementation of ETAT (emergency triage assessment and treatment) in a central hospital in Malawi. Arch Dis Child.

[25] Johansson EW, Lindsjö C, Weiss DJ, Nsona H, Selling KE, Lufesi N, Hildenwall H (2020). Accessibility of basic paediatric emergency care in Malawi: analysis of a national facility census. BMC Public Health.

[26] Development R (2013). Core team: R: a language and environment for statistical computing.

[27] Campbell H, Duke T, Weber M, English M, Carai S, Tamburlini G (2008). Global initiatives for improving hospital care for children: state of the art and future prospects. Pediatrics.

[28] Mills R, Seager E, Harris C, Hiwa T, Blackstock S, Pumphrey J, Kennedy N, Langton J (2017). G321(P) the causes of paediatric inpatient deaths in Malawi. Arch Dis Child.

[29] Robertson SK, Manson K, Fioratou E (2018). IMCI and ETAT integration at a primary healthcare facility in Malawi: a human factors approach. BMC Health Serv Res.

[30] Font F, Alonso González M, Nathan R, Kimario J, Lwilla F, Ascaso C, Tanner M, Menéndez C, Alonso PL (2001). Diagnostic accuracy and case management of clinical malaria in the primary health services of a rural area in South-Eastern Tanzania. Tropical Med Int Health.

[31] Redd SC, Bloland PB, Kazembe PN, Patrick E, Tembenu R, Campbell CC (1992). Usefulness of clinical case-definitions in guiding therapy for African children with malaria or pneumonia. Lancet.

[32] Hall CB, Weinberg GA, Iwane MK, Blumkin AK, Edwards KM, Staat MA, Auinger P, Griffin MR, Poehling KA, Erdman D (2009). The burden of respiratory syncytial virus infection in young children. N Engl J Med.

[33] Graham SM, English M, Hazir T, Enarson P, Duke T (2008). Challenges to improving case management of childhood pneumonia at health facilities in resource-limited settings. Bull World Health Organ.

[34] World Health Organization (2013). Pocket book of hospital care for children: guidelines for the management of common illnesses with limited resources.

[35] Duke T (2014). Pneumonia and bronchiolitis in developing countries. Arch Dis Child.

[36] Govender I, Steyn C, Maricowitz G, Clark CC, Tjale MC (2018). A primary care physician’s approach to a child with meningitis. South Afr J Infect Dis.

[37] Shaker R, Fayad D, Dbaibo G (2018). Challenges and opportunities for meningococcal vaccination in the developing world. Hum Vacc Immunotherapeutics.

[38] Petti CA, Polage CR, Quinn TC, Ronald AR, Sande MA (2006). Laboratory medicine in Africa: a barrier to effective Health care. Clin Infect Dis.

[39] Zhang HL, Omondi MW, Musyoka AM, Afwamba IA, Swai RP, Karia FP, Muiruri C, Reddy EA, Crump JA, Rubach MP (2016). Challenges of maintaining good clinical laboratory practices in low-resource settings: a Health program evaluation framework case study from East Africa. Am J Clin Pathol.

